# Will driverless cars be good for us? Now is the time for public health to act together with urban and transport planning

**DOI:** 10.7189/jogh-09-020303

**Published:** 2019-12

**Authors:** Angela Curl, Helen Fitt

**Affiliations:** 1 Department of Population Health, University of Otago Christchurch, Christchurch, New Zealand; 2 Department of Geography, University of Canterbury, Christchurch, New Zealand; * Equal authorship

A new “drug” currently undergoing development could treat at least two persistent global health conditions:

- mortality and morbidity from road traffic collisions,
- loneliness and exclusion for less mobile individuals.

This drug is widely known as the “driverless car”. Unfortunately, side effects may be substantial and there is risk of dependency, as with the private car [1].

Claims that driverless cars will considerably reduce transport related non-communicable diseases (NCDs) and injury are prevalent and compelling. Road crashes are the eighth leading cause of death globally and result in 1.35 million deaths per year [2]. Up to 90% of traffic collisions are attributed to driver error [3], which could be eliminated by vehicle automation. Additionally, experiences of social isolation, exclusion, and loneliness – that can occur as a result of limited transport options – pose mortality risks comparable to smoking [4]. Driverless cars may enhance the mobility of non-drivers, thus facilitating social connection and well-being, especially in ageing populations. The health sector should, therefore, be interested in the potential of the driverless car ‘drug’ to address some global health concerns.

However, potential repercussions of driverless cars for other health outcomes have been the subject of little research [5]. These can be illuminated by lessons from historical transport transitions. The widespread adoption of private cars led to economic and social benefits but also extensive environmental and health costs. It is widely accepted that dispersed urban environments and associated car dependence in many countries contribute substantially to the global burden of NCDs and injury through exposure to risks such as physical inactivity, noise, pollution, and inaccessibility for non-drivers. Exposure to such risks results in conditions including respiratory illnesses, cardiovascular conditions, type II diabetes, cancer, road trauma and poor mental well-being [6-8]. An adoption of driverless cars will, likewise, have implications for travel behaviour and urban environments with pervasive impacts on population health.

There is currently little discussion of how a transition to automation will impact global regions differently. The health impacts of automation will vary globally according to prevailing levels of car reliance, conditions of the road network, and availability and affordability of new technologies. As a result, the global north may be first to benefit from improved safety, while 93% of road traffic deaths are in low to middle income countries [2]. Already, transport planners do not expect that safety benefits will be evenly distributed among population groups [9]. Transport technologies have the potential, therefore, to alter trajectories of development and increase or decrease global inequalities.

Many claims about the potential of driverless cars to treat global health concerns rely on assumptions of full automation, electric vehicles, and shared transport, which are not guaranteed. Partial automation could result in an increase in risky driver behaviour [5] and deteriorations in road safety; resistance to shared transport could result in growing traffic volumes; and continued use of internal combustion engines could result in worsening pollution. Even if full automation, electrification, and sharing eventuate, this will not be instantaneous and the period of transition could include problematic dynamics.

Recent years have seen increased recognition of the role urban environments play in influencing a plethora of health outcomes [6-8]. This has highlighted a need to focus on the social and structural determinants of health and has increased dialogue between urban and transport planners and public health professionals. In the face of forthcoming changes to transport systems and cities, this dialogue is important as ever.

If driverless cars are a given, urban and transport planners have an important role to play in how effective this technological development is in addressing global health inequalities. However, good urban and transport planning are also preventative health measures. Urban planning which supports active modes of transport and improves local accessibility can help prevent many of the health issues which driverless cars are claimed to “treat”.

We currently have a (time-limited) opportunity to shape future social determinants of health through transport system transitions. Meeting the challenges ahead requires action in at least three domains. First, we need to take a preventative approach to non-communicable diseases including those related to transport and urban environments, rather than waiting to treat the problem, as we are now doing in response to the growth of private car use. Second, we need to increase collaboration between urban planners and health professionals. Health professionals can bring considerable expertise to urban and transport planning, including through health impact assessments. Third, we need to encourage a focus on health and well-being outcomes – rather than only indicators of economic progress – when assessing emerging technologies and population health. These three priority actions are general strategies for encouraging development of healthy transport systems.

**Figure:**
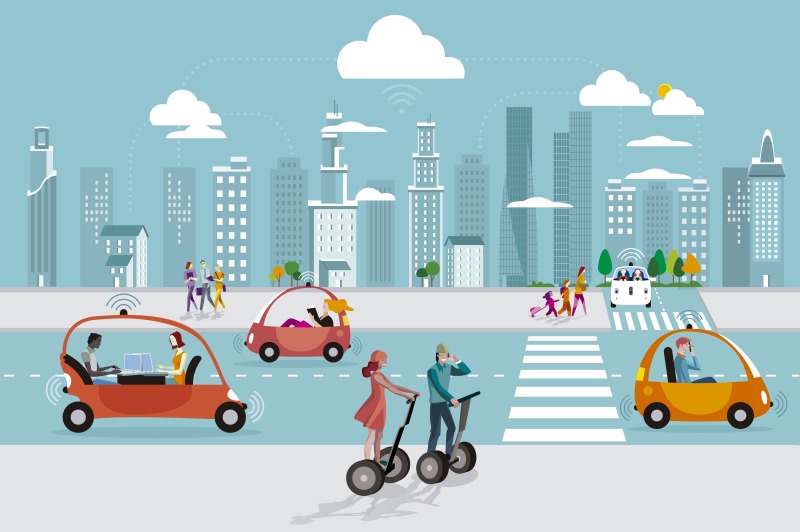
Photo: Driverless autonomous car in the city (by Jesussanz, used with permission).

Tackling NCDs is a major global health concern for the foreseeable future [10]. There is now a fleeting opportunity for public health professionals to engage in transport technology debates. Driverless cars may be an enticing drug to treat some current public health concerns, but only if urban and transport planners and public health professionals take a concerted and proactive approach to ensuring their effectiveness, managing the potential side effects and preventing unnecessary use where alternatives, such as creating walkable communities, already exist.
